# Supplementary material for: A universal dual mechanism immunotherapy for the treatment of influenza virus infections
Source: Nat Commun. 2020 Nov 5;11:5597. doi: 10.1038/s41467-020-19386-5 (PMC7645797; doi:10.1038/s41467-020-19386-5)
Supplement: Supplementary file 1 — Supplementary Information [file 41467_2020_19386_MOESM1_ESM.pdf]

Supplementary Information

A universal dual mechanism immunotherapy for the treatment of influenza virus infections

Xin Liu,[1,2] Boning Zhang,[1,2] Yingcai Wang,[1,2] Hanan S. Haymour,[1,2] Fenghua Zhang,[1,2] Le-cun Xu,[3] Madduri Srinivasarao,[1,2] Philip S. Low[1,2*]

Affiliations:
[1]Department of Chemistry, Purdue University, West Lafayette, IN 47907, USA.

[2]Purdue Institute for Drug Discovery, Purdue University, West Lafayette, IN 47907, USA.

[3]Endocyte Inc., 3000 Kent Ave, West Lafayette, IN 47906, USA.

*Correspondence: plow@purdue.edu

Supplementary Methods
Supplementary Table 1-2
Supplementary Figure 1 to 14

Supplementary Methods

Synthesis materials: Azido-dPEG®$_3$-amine was purchased from Quanta BioDesign (Plain City, OH). Amino-PEG$_5$-amine was purchased from Combi-Blocks (San Diego, CA). DBCO-NHS (dibenzocyclooctyne-N-hydroxysuccinimidyl ester), DBCO-amine and DBCO-acid were obtained from Conju-Probe (San Diego, CA). NHS-rhodamine was purchased from Thermo Fisher Scientific (Waltham, MA). All other chemicals and reagents were purchased from Sigma-Aldrich (St. Louis, MO). Compounds were purified by flash column chromatography (Teledyne CombiFlash Rf+Lumen system) or preparative high-performance liquid chromatography (Prep-HPLC, Agilent 1200 series semipreparatory HPLC). The mass spectra of compounds were obtained by liquid chromatography–mass spectrometry (LC-MS, Agilent 1220 series LC-coupled 6130 quadrupole MS).

Synthesis of zanamivir-related influenza neuraminidase targeting ligand (Supplementary Fig. 11)

Zanamivir derivative 1 was prepared from sialic acid according to previously reported methods [1-3].

Compound 2. To a solution of zanamivir derivative **1** (1.0 g, 1.33 mmol) in pyridine (12.0 mL) was added azido-dPEG®$_3$-amine (871 mg, 3.99 mmol) and 4-(dimethylamino)pyridine (DMAP) (244 mg, 2.0 mmol). The reaction mixture was stirred at room temperature (rt) overnight and then concentrated under reduced pressure. The crude product was purified by flash column chromatography on a Teledyne CombiFlash Rf+ Lumen (silica gel column, 0-100% EtOAc in hexanes) to give **2** as a yellow powder (588 mg, 53%). LC-MS $[M + H]^+$ = 831.3. ^{1}H NMR (500 MHz, DMSO-d6) δ: 1.24 (s, 3H), 1.28 (s, 3H), 1.37 (s, 9H), 1.43 (s, 9H), 1.71 (s, 3H), 3.03 (m,

1H), 3.33 (m, 7H), 3.53 (m, 8H), 3.71 (s, 3H), 3.85 (dd, J = 8.9, 5.5 Hz, 1H), 3.93 (dd, J = 13.6, 6.2 Hz, 1H), 4.02 (dd, J = 8.9, 6.0 Hz, 1H), 4.22 (q, J = 5.9 Hz, 1H), 4.34 (d, J = 10.4 Hz, 1H), 4.78 (d, J = 8.7 Hz, 1H), 5.14 (d, J = 6.0 Hz, 1H), 5.79 (dd, J = 5.9, 2.4 Hz, 1H), 7.18 (t, J = 5.8 Hz, 1H), 7.94 (d, J = 9.0 Hz, 1H), 8.16 (d, J = 7.6 Hz, 1H), 11.33 (s, 1H).

Compound 3. Compound **2** (530 mg, 0.64 mmol) was dissolved in THF (5 mL) and treated dropwise with 5 ml 1 M NaOH (aq). The reaction mixture was stirred at rt for 1 h at which time LC-MS analysis revealed that the ester was completely converted to the acid. The reaction mixture was neutralized by adding Dowex® 50WX8 (H^+) resin, filtered, and concentrated under reduced pressure. The white crude product **3** was used directly for the next step without purification. LC-MS $[M + H]^+$ = 817.3.

Zanamivir targeting ligand 4. To crude compound **3** was added trifluoroacetic acid (TFA) (8 mL). The solution was stirred open to air for 2 h at rt until the reaction was completed as demonstrated by LC-MS. TFA was removed by rotary evaporation under reduced pressure and the product was purified by Prep-HPLC on a C18 column (0-50% acetonitrile in 1% TFA) to give **4** as a white powder (290 mg). The total yield over the 2 steps was 79%. LC-MS $[M + H]^+$ = 577.2. ^{1}H NMR (500 MHz, DMSO-d6) δ: 1.77 (s, 3H), 3.03 (q, J = 6.4 Hz, 2H), 3.22 (dd, J = 11.4, 7.4 Hz, 1H), 3.34 (m, 1H), 3.39 (m, 4H), 3.49 (m, 6H), 3.56 (m, 6H), 3.78 (m, 1H), 3.97 (q, J = 9.3 Hz, 1H), 4.30 (m, 1H), 4.37 (m, 1H), 4.81 (dd, J = 9.2, 2.3 Hz, 1H), 5.68 (t, J = 2.8 Hz, 1H), 7.08 (s, 1H), 7.17 (dd, J = 10.0, 4.3 Hz, 1H), 7.28 (s, 1H), 7.62 (d, J = 8.8 Hz, 1H), 7.98 (d, J = 9.2 Hz, 1H).

Synthesis of zan-DNP (Supplementary Fig. 12)

Compound 6. To a solution of 1-chloro-2,4-dinitrobenzene (90 mg, 0.45 mmol) and amino-PEG_5-amine (250 mg, 0.89 mmol) dissolved in EtOH (6 mL) was added triethylamine (TEA) (250 µl, 1.79 mmol). The reaction mixture was heated to reflux for 5h, cooled and concentrated under reduced pressure. The crude mixture was purified by flash column chromatography on Teledyne CombiFlash Rf+ Lumen (C18 column, 0-100% acetonitrile in aqueous 10 mM NH_4OAc pH7 buffer) to yield **6** as a yellow oil (159 mg, 80%). LC-MS $[M + H]^+$ =447.2.

Compound 7. To a solution of DBCO-NHS (100 mg, 0.25 mmol) and compound 6 (110 mg, 0.25 mmol) dissolved in DMSO (6 mL) was added N,N-diisopropylethylamine (DIPEA) (500 µl, 2.87 mmol) dropwise. The reaction mixture was stirred under rt for 30 min and then purified directly by flash column chromatography on Teledyne CombiFlash Rf+ Lumen (C18 column, 0-100% acetonitrile in aqueous 10 mM NH_4OAc pH7 buffer) to afford **7** as a yellow oil (130 mg, 71%). LC-MS $[M + H]^+$ = 734.2.

Zan-DNP 8. Zanamivir targeting ligand **4** (52 mg, 0.09 mmol) and compound **7** (82 mg, 0.11 mmol) were dissolved in DMSO (5 mL) and stirred at rt while monitoring the reaction progress by LC-MS. After the complete consumption of zanamivir targeting ligand **4** (0.5 h), the reaction mixture was purified by Prep-HPLC on a C18 column (0-50% acetonitrile in aqueous 10 mM NH_4OAc pH7 buffer) to yield **8** as a yellow powder (87 mg, 74%). LC-MS $[M + H]^+$= 1310.4.

Synthesis of zan-rhodamine (Supplementary Fig. 13)

Compound 10. To a solution of NHS-Rhodamine **9** (15.0 mg, 0.03 mmol) and DBCO-amine (15.5 mg, 0.06 mmol) dissolved in DMSO (2 mL) was added DIPEA (50 µl, 0.29 mmol). The reaction mixture was stirred at rt for 1h and then purified directly by Prep-HPLC on a C18 column (0-100%

acetonitrile in aqueous 10 mM NH_4OAc pH7 buffer) to give **10** as a red powder (6.0 mg, 32%). LC-MS $[M + H]^+$ = 689.3

Zan-rhodamine 11. Zanamivir targeting ligand **4** (10.2 mg, 0.02 mmol) and compound **10** (6.0 mg, 0.01 mmol) were dissolved in DMSO (2 mL) and stirred at rt. The reaction progress was monitored by LC-MS. After consumption of compound **10** (1 h), the reaction mixture was purified by Prep-HPLC on a C18 column (0-100% acetonitrile in aqueous 10 mM NH_4OAc pH7 buffer) to yield **11** as a red powder (3.2 mg, 29%). LC-MS $[M + H]^+$ = 1265.3.

Synthesis of zan-^{99m}Tc (Supplementary Fig. 14)

Compound 12, a peptide chelator of ^{99m}Tc comprising a sequence β-L-diaminopropionic acid, L-aspartic acid (L-Asp) and L-cysteine (L-Cys), was prepared as described previously in our lab [4,5].

Compound 13. To a peptide synthesis vessel containing compound **12** bound to H-Cys(Trt)-2-ClTrt resin (370 mg, 0.11 mmol, resin loading: 0.3 mmol/g) was added DBCO acid (100 mg, 0.33 mmol), (benzotriazol-1-yloxy)tripyrrolidinophosphonium hexafluorophosphate (PyBop) (115 mg, 0.22 mmol), DMF (3 mL) and DIPEA (58 µl, 0.33 mmol) and the suspension was bubbled with argon overnight. The product was then cleaved from the resin using a cocktail cleavage solution consisting of 92.5 % TFA, 2.5% triisopropylsilane (TIPS), 2.5% ethanethiol (EtSH) and 2.5% H_2O, and the released compound was precipitated in diethyl ether, dried under vacuum, and purified by Prep-HPLC on a C18 column (0-100% acetonitrile in aqueous 10 mM NH_4OAc pH5 buffer) to afford **13** as a white powder (47 mg, 70%). LC-MS $[M + H]^+$ = 610.2.

zan-^{99m}Tc 14. Zanamivir-related targeting ligand **4** (17 mg, 0.03 mmol) and compound **7** (27.4 mg, 0.05 mmol) were dissolved in DMSO (3 mL) and stirred at rt for ~1h until zanamivir targeting ligand **4** was consumed. The product was then purified by Prep-HPLC on a C18 column (0-50% acetonitrile in aqueous 10 mM NH_4OAc pH7 buffer) to give **14** as a white powder (23 mg, 64%). LC-MS $[M + H]^+$ = 1186.3.

figure		P value
Fig. 3e	difference between organe bar and black bar (hemagglutinin+ cells)	0.0011
	difference between green bar and black bar (hemagglutinin+ cells)	<0.0001
	difference between purple bar and black bar (hemagglutinin+ cells)	<0.0001
	difference between organe bar and black bar (anti-DNP antibody+ cells)	0.0125
	difference between organe bar and purple bar (anti-DNP antibody+ cells)	0.003
	difference between organe bar and green bar (anti-DNP antibody+ cells)	0.002
Fig. 4a	0.5 µmol/kg (0.7 mg/kg) zan-DNP	<0.0001
	0.5 µmol/kg (0.2 mg/kg) zanamivir	0.029
	0.5 µmol/kg (0.1 mg/kg) DNP	0.7494
	0.5 µmol/kg zanamivir + DNP	0.023
	0.5 µmol/kg (0.7 mg/kg) zan-DNP (unimmunized mice)	0.0002
Fig. 4b	48 hpi, 0.5 µmol/kg (0.7 mg/kg) zan-DNP	0.002
	72 hpi, 0.5 µmol/kg (0.7 mg/kg) zan-DNP	0.002
	96 hpi, 0.5 µmol/kg (0.7 mg/kg) zan-DNP	0.0273
	48 hpi, 0.5 µmol/kg (0.2 mg/kg) zanamivir	0.0437
	48 hpi, 0.5 µmol/kg (0.7 mg/kg) zan-DNP (unimmunized mice)	0.0273
Fig. 4c	1.5 µmol/kg (2.0 mg/kg) zan-DNP (upper left panel)	0.0023
	0.5 µmol/kg (0.7 mg/kg) zan-DNP (upper left panel)	0.0023
	1.5 µmol/kg (0.5 mg/kg) zanamivir (upper left panel)	0.008
	1.5 µmol/kg (2.0 mg/kg) zan-DNP (upper right panel)	0.0016
	0.5 µmol/kg (0.7 mg/kg) zan-DNP (upper right panel)	0.0016
	1.5 µmol/kg (0.5 mg/kg) zanamivir (upper right panel)	0.0158
	1.5 µmol/kg (2.0 mg/kg) zan-DNP (lower left panel)	0.0025
	0.5 µmol/kg (0.7 mg/kg) zan-DNP (lower left panel)	0.0025
	1.5 µmol/kg (0.5 mg/kg) zanamivir (lower left panel)	0.1694
	1.5 µmol/kg (2.0 mg/kg) zan-DNP (lower right panel)	<0.0001
	0.5 µmol/kg (0.7 mg/kg) zan-DNP (lower right panel)	<0.0001
	1.5 µmol/kg (0.5 mg/kg) zanamivir (lower right panel)	0.0001
Fig. 5e	4.5 µmol/kg (5.9 mg/kg) zan-DNP (upper left panel)	0.0027
	1.5 µmol/kg (2.0 mg/kg) zan-DNP (upper left panel)	0.0027
	4.5 µmol/kg (1.5 mg/kg) zanamivir (upper left panel)	0.1336
	4.5 µmol/kg (5.9 mg/kg) zan-DNP (upper right panel)	0.0019
	1.5 µmol/kg (2.0 mg/kg) zan-DNP (upper right panel)	0.0019
	4.5 µmol/kg (1.5 mg/kg) zanamivir (upper right panel)	0.7198
	4.5 µmol/kg (5.9 mg/kg) zan-DNP (lower left panel)	0.0025
	1.5 µmol/kg (2.0 mg/kg) zan-DNP (lower left panel)	0.0025
	4.5 µmol/kg (1.5 mg/kg) zanamivir (lower left panel)	0.6361
	4.5 µmol/kg (5.9 mg/kg) zan-DNP (lower right panel)	<0.0001
	1.5 µmol/kg (2.0 mg/kg) zan-DNP (lower right panel)	<0.0001
	4.5 µmol/kg (1.5 mg/kg) zanamivir (lower right panel)	0.3532
Fig. 6a	1.5 umol/kg (2.0 mg/kg) zan-DNP (IN), 10 mg/kg anti-DNP antibody (IV)	0.0027
	1.5 umol/kg (2.0 mg/kg) zan-DNP (IN), 3 mg/kg anti-DNP antibody (IV)	0.0027
	1.5 umol/kg (2.0 mg/kg) zan-DNP (IN), 1 mg/kg anti-DNP antibody (IV)	0.0027
	1.5 umol/kg (2.0 mg/kg) zan-DNP (IN), w/o anti-DNP antibody (IV)	0.0143
	1.5 umol/kg (0.5 mg/kg) zanamivir (IN), 10 mg/kg anti-DNP antibody (IV)	0.0495
Fig. 6b	1.5 umol/kg (2.0 mg/kg) zan-DNP (IN), 10 mg/kg anti-DNP antibody (IV) (upper left panel)	<0.0001
	1.5 umol/kg (0.5 mg/kg) zanamivir (IN), 10 mg/kg anti-DNP antibody (IV) (upper left panel)	0.0495
	1.5 umol/kg (2.0 mg/kg) zan-DNP (IN), 10 mg/kg anti-DNP antibody (IV) (upper right panel)	<0.0001
	1.5 umol/kg (0.5 mg/kg) zanamivir (IN), 10 mg/kg anti-DNP antibody (IV) (upper right panel)	0.8858
	1.5 umol/kg (2.0 mg/kg) zan-DNP (IN), 10 mg/kg anti-DNP antibody (IV) (lower left panel)	0.0005
	1.5 umol/kg (0.5 mg/kg) zanamivir (IN), 10 mg/kg anti-DNP antibody (IV) (lower left panel)	0.4878
Supplementary Fig. 7a	1.5 µmol/kg (2.0 mg/kg) zan-DNP	<0.0001
	0.5 µmol/kg (0.7 mg/kg) zan-DNP	<0.0001
	0.17 µmol/kg (0.2 mg/kg) zan-DNP	<0.0001
	13.5 µmol/kg (4.5 mg/kg) zanamivir	<0.0001
	4.5 µmol/kg (1.5 mg/kg) zanamivir	<0.0001
	1.5 µmol/kg (0.5 mg/kg) zanamivir	<0.0001
	0.5 µmol/kg (0.2 mg/kg) zanamivir	0.0031
Supplementary Fig. 7b	48 hpi, 1.5 µmol/kg (2.0 mg/kg) zan-DNP	0.002
	72 hpi, 1.5 µmol/kg (2.0 mg/kg) zan-DNP	0.002
	96 hpi, 1.5 µmol/kg (2.0 mg/kg) zan-DNP	0.0854
	48 hpi, 1.5 µmol/kg (0.5 mg/kg) zanamivir	0.0158
	48 hpi, 1.5 µmol/kg (2.0 mg/kg) zan-DNP (unimmunized mice)	0.0052
Supplementary Fig. 7c	1.5 µmol/kg (2.0 mg/kg) zan-DNP, every day, 5 times	0.0023
	1.5 µmol/kg (2.0 mg/kg) zan-DNP, every other day, 3 times	0.0023
	1.5 µmol/kg (2.0 mg/kg) zan-DNP, 1 time	0.0023
Supplementary Fig. 8a	difference between zan-DNP and PBS	0.0141
	difference between zan-DNP and zanamivir	0.0445
	difference between zanamivir and PBS	0.0146
Supplementary Fig. 8b	difference between zan-DNP and PBS	0.0002
	difference between zan-DNP and zanamivir	0.0004
	difference between zanamivir and PBS	0.06
Supplementary Fig. 10a	4.5 µmol/kg (5.9 mg/kg) zan-DNP	0.0025
	1.5 µmol/kg (2.0 mg/kg) zan-DNP	0.0025
	0.5 µmol/kg (0.7 mg/kg) zan-DNP	0.0025
	40.5 µmol/kg (13.5 mg/kg) zanamivir	0.0025
	13.5 µmol/kg (4.5 mg/kg) zanamivir	0.0025
	4.5 µmol/kg (1.5 mg/kg) zanamivir	0.0025
Supplementary Fig. 10b	0.5 µmol/kg (0.7 mg/kg) zan-DNP	0.002
	0.5 µmol/kg (0.2 mg/kg) zanamivir	0.1112
	0.5 µmol/kg (0.1 mg/kg) DNP	0.8566
	0.5 µmol/kg zanamivir + DNP	0.7756
	0.5 µmol/kg (0.7 mg/kg) zan-DNP (unimmunized mice)	0.2908

Supplementary Table 1. P values of the statistical tests

name	sequence
influenza matrix (M) gene (forward primer)	5’-AAGACCAATCCTGTCACCTCTGA -3’
influenza matrix (M) gene (reverse primer)	5’-CAAAGCGTCTACGCTGCAGTCC-3’
influenza matrix (M) gene (probe)	5’-(FAM)-TTTGTGTTCACGCTCACCGT-(TAMRA)-3’

Supplementary Table 2. Primers used in real time RT-PCR (quantitation of lung viral titers)

a

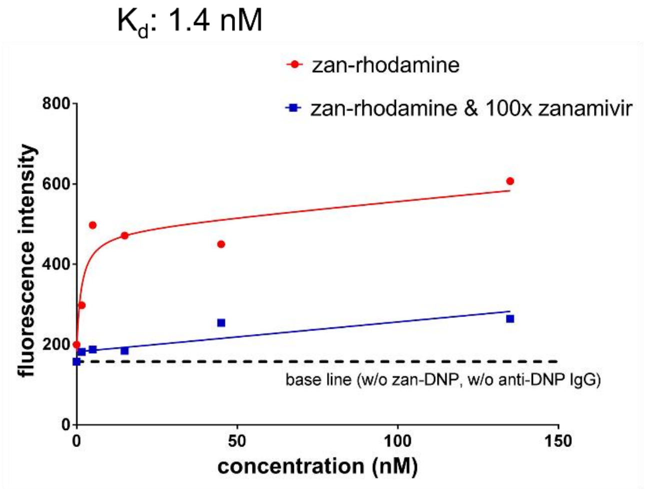

b

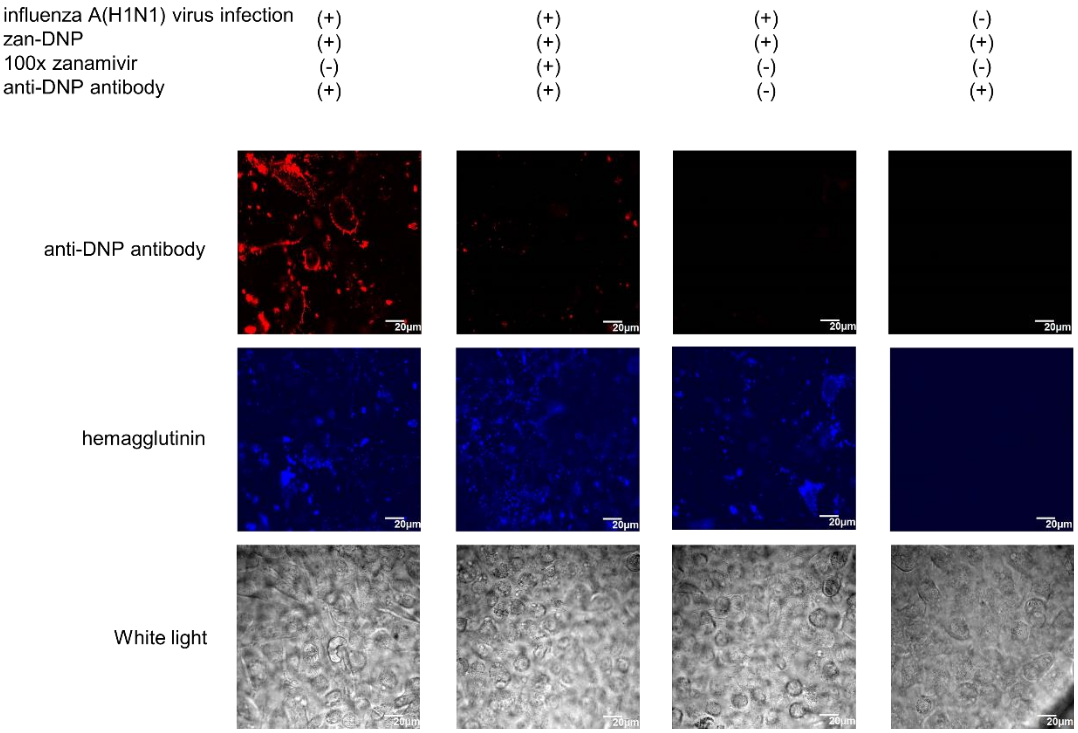

Supplementary Figure 1. In vitro binding affinity and antibody recruitment assays performed using virus-infected primary human lung cells. (**a**) Binding of zan-rhodamine (direct binding curve) to neuraminidase expressed on normal human bronchial epithelial (NHBE) cells (grown at air-liquid interface) infected with influenza virus A/Wisconsin/629-D00015/2009 (H1N1)pdm09 (*n*=2). (**b**) Confocal micrographs of binding of human anti-DNP IgG (visualized by goat anti-human IgG-PE) to the surface of A/Wisconsin/629-D00015/2009 (H1N1)pdm09 virus-infected NHBE cells (grown at air-liquid interface) mediated by zan-DNP (*n*=3). Hemagglutinin staining is shown in blue, and anti-human IgG-PE staining is shown in red. All the virus-infected cells were used 36h post-infection for the binding and confocal imaging studies.

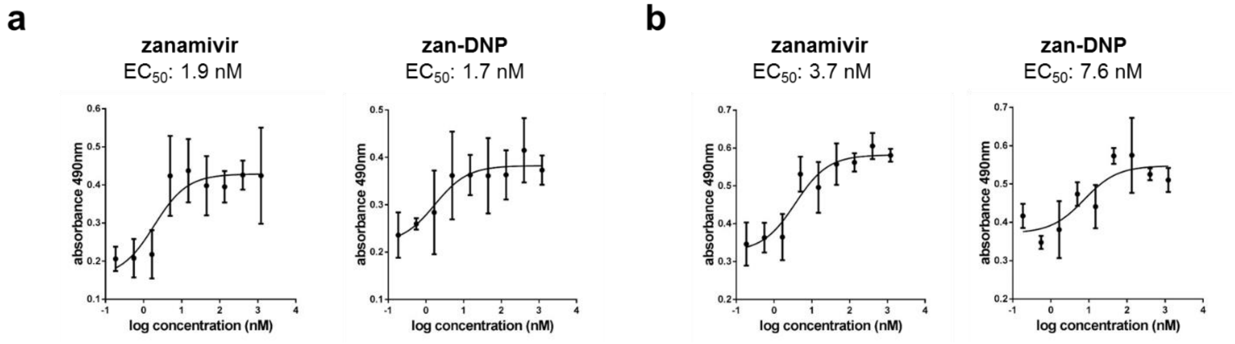

Supplementary Figure 2. Dose-response curves of in-vitro anti-viral activity of zanamivir (left panel) and zan-DNP (right panel) for influenza virus A/Puerto Rico/8/1934 (H1N1) (**a**) and A/Aichi/2/1968 (H3N2) (**b**) virus-infected MDCK cells. Cell viability was quantitated by measuring absorbance using a CellTiter 96 assay. Data are presented as mean values +/- SD (*n*=3).

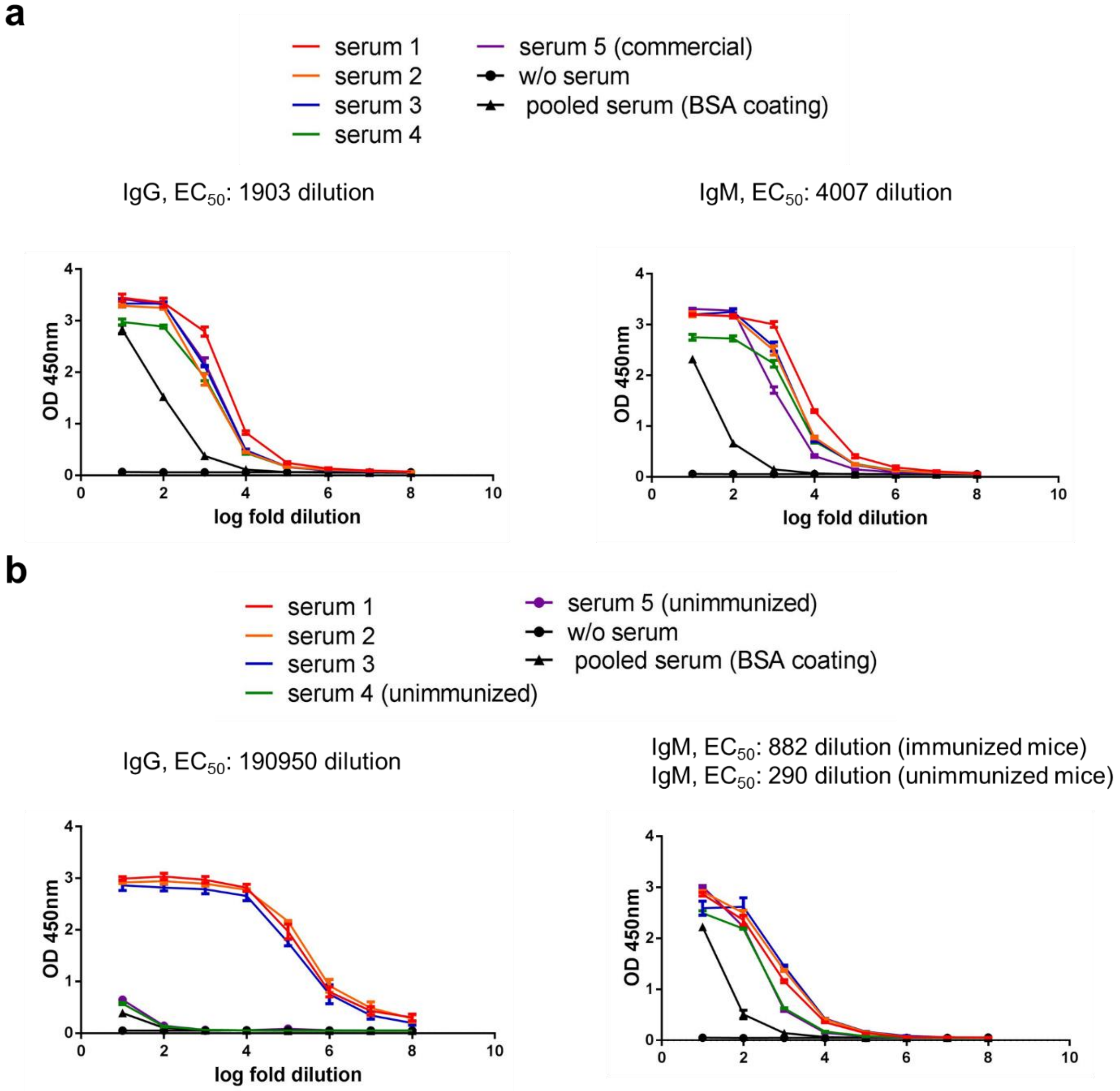

Supplementary Figure 3. Analysis of the natural anti-DNP antibody titers in human serum and DNP-KLH immunized mice. The titers of anti-DNP antibodies were measured by ELISA and plotted as average absorbance at 450 nm versus log serum dilution. (**a**) Titer of human anti-DNP IgG (left panel) and anti-DNP IgM (right panel). Serum samples 1-4 were collected from 4 healthy volunteers; serum 5 was a purchased pooled human serum sample (Valley Biomedical). (**b**) Titer of DNP-KLH immunized mouse anti-DNP IgG (left panel) and anti-DNP IgM (right panel). Serum samples 1-3 were collected from 3 DNP-KLH immunized mice; serum samples 4-5 were collected from 2 unimmunized mice. Data are presented as mean values +/- SD. (*n*=3).

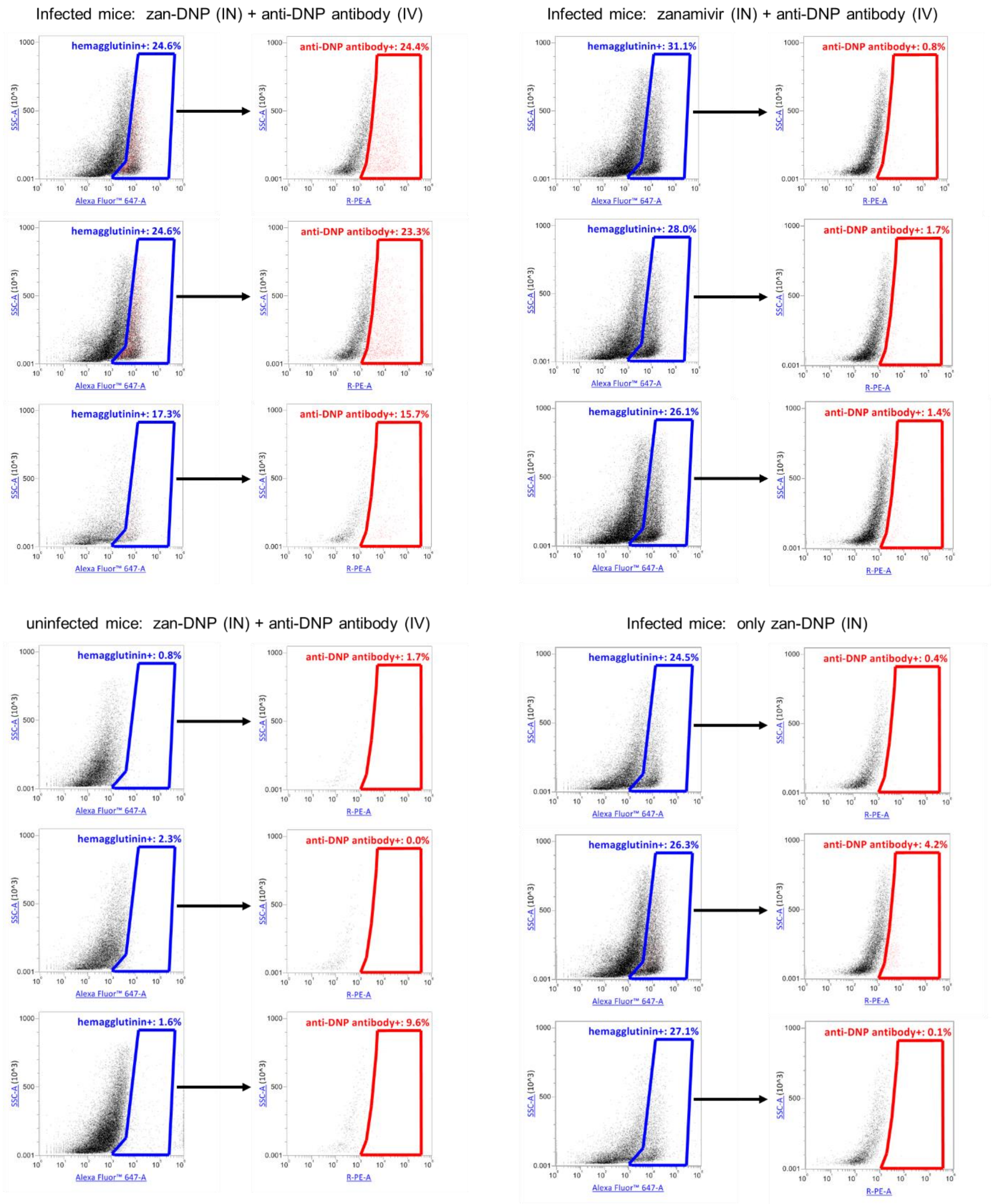

Supplementary Figure 4. Flowcytometry analyses of recruitment of anti-DNP antibodies to virus-infected cells mediated by zan-DNP in vivo. A/California/07/2009 (H1N1)pdm09 virus-infected mice (3 mice/group) were first given zan-DNP and anti-DNP antibodies. The cells digested from the lung of virus-infected/uninfected mice were stained first with anti-hemagglutinin antibodies and then labeled with dye conjugated secondary antibodies against anti-hemagglutinin antibodies and anti-DNP antibodies (*n*=3).

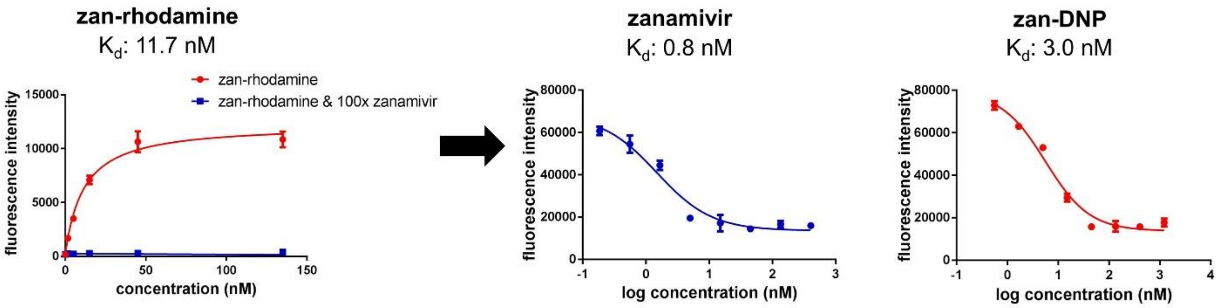

Supplementary Figure 5. Binding of zan-rhodamine (saturation binding curve, left panel), zanamivir (competitive binding curve, central panel) and zan-DNP (competitive binding curve, right panel) for N1 neuraminidase (expressed on N1 neuraminidase transduced HEK 293 cells). The coding sequence of N1 neuraminidase for transduction was from strain A/Puerto Rico/8/1934 (H1N1); Data are presented as mean values +/- SD (*n*=3).

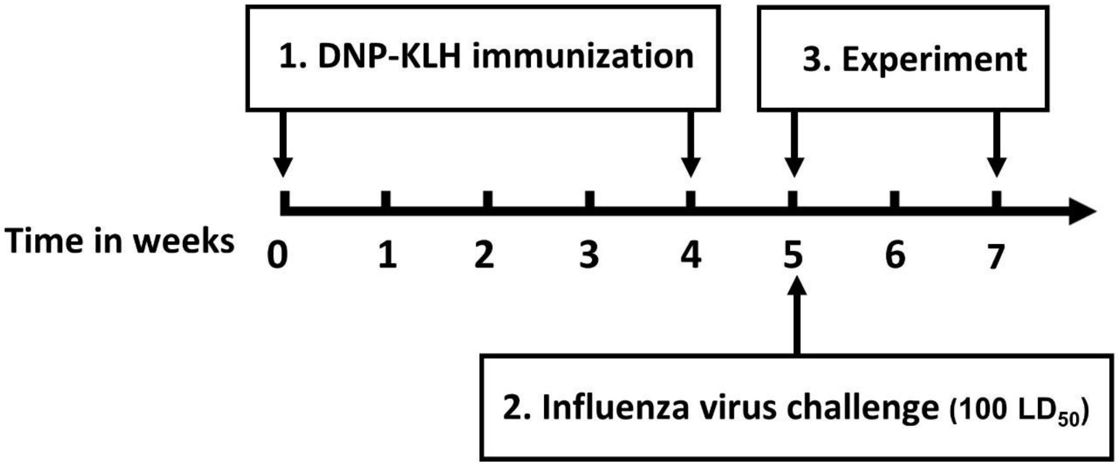

Supplementary Figure 6. General procedure for mouse therapy studies. 3 to 4 week old BALB/c mice were first immunized with DNP-KLH and then challenged 5 weeks later with 100x MLD_{50} of infleunza A(H1N1) or A(H3N2) virus by intranasal inoculation. During the following two weeks, mice were treated with different test articles (zan-DNP, zanamivir, DNP or PBS) according to the indicated dosing regimens. Mice were weighed and monitored daily for 14 days after infection. Mice were counted as dead when they lost either 25% of their initial weight or became moribund.

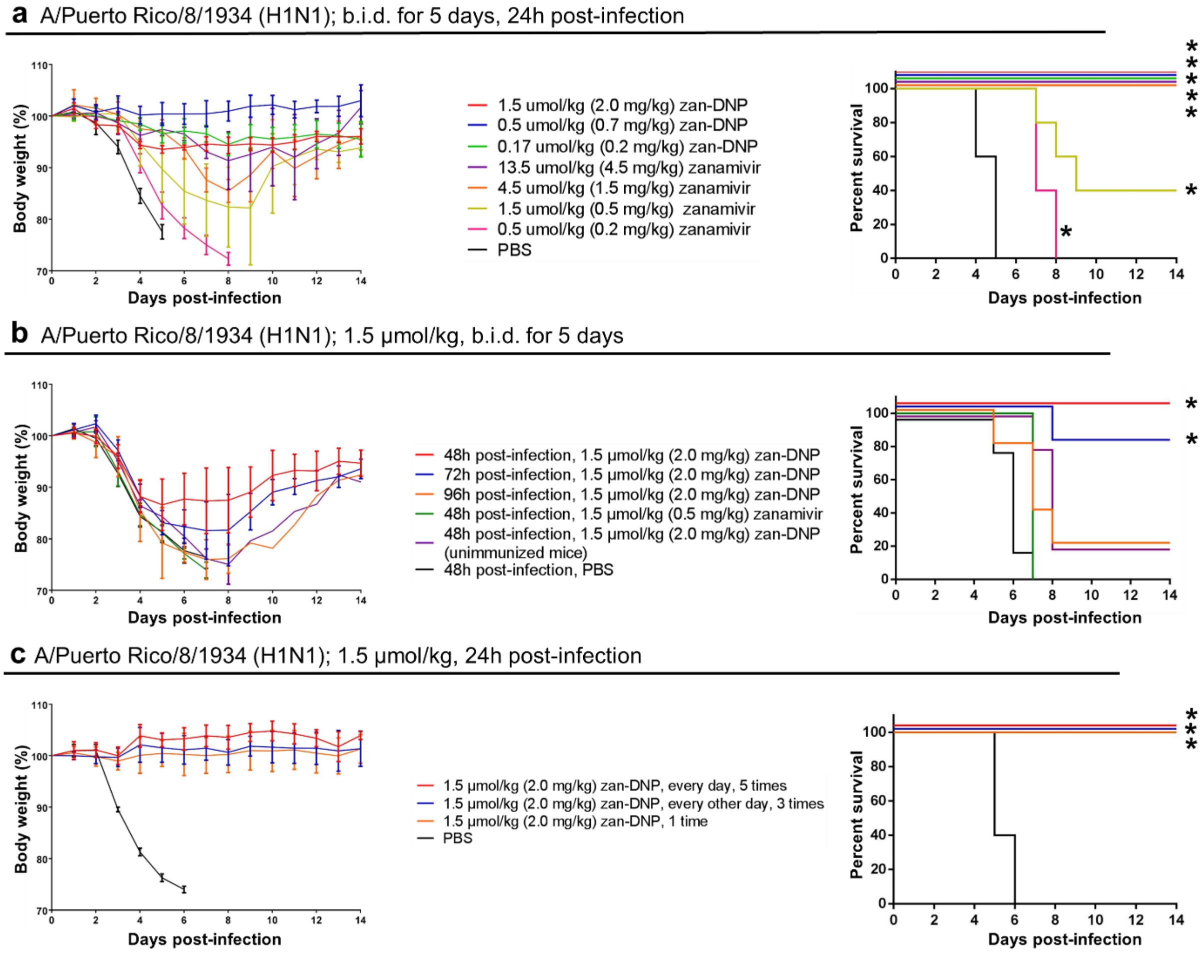

Supplementary Figure 7. In vivo therapeutic efficacy of zan-DNP (intranasal administration). DNP-KLH immunized BALB/c mice were challenged with 100x MLD_{50} of influenza virus A/Puerto Rico/8/1934 (H1N1). Mice were treated with the indicated test articles by intranasally administration using the dosing regimens indicated in the figure. (**a**) Dose ranging study (5 mice/group). (**b**) Delayed-start-to-treat study using 1.5 μmol/kg zan-DNP/zanamivir (5 mice/group). (**c**) Dose frequency study (5 mice/group). Statistical differences between PBS and drug treatment groups were determined by two-sided log-rank test (*$P < 0.005$, see exact P values in Supplementary Table 1). Body weight change (%) are presented as mean values +/- SD.

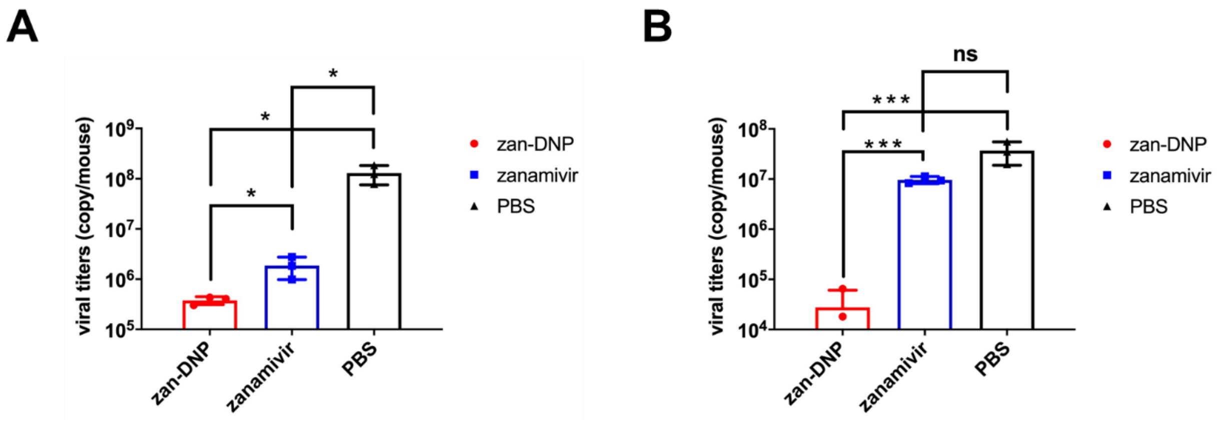

Supplementary Figure 8. Viral titers in the lungs of DNP-KLH immunized mice following treatment with a single dose of 1.5 µmol/kg zan-DNP/zanamivir or PBS. (**a**) Lung viral titers measured 3 days post-infection. (**b**) Lung viral titers measured 5 days post-infection. Statistical difference of lung viral titers between any two groups was analyzed by two-sided t test (**P* < 0.05, ***P* < 0.01, ****P* < 0.001, see exact P values in Supplementary Table 1). Data are presented as mean values +/- SD (*n*=3).

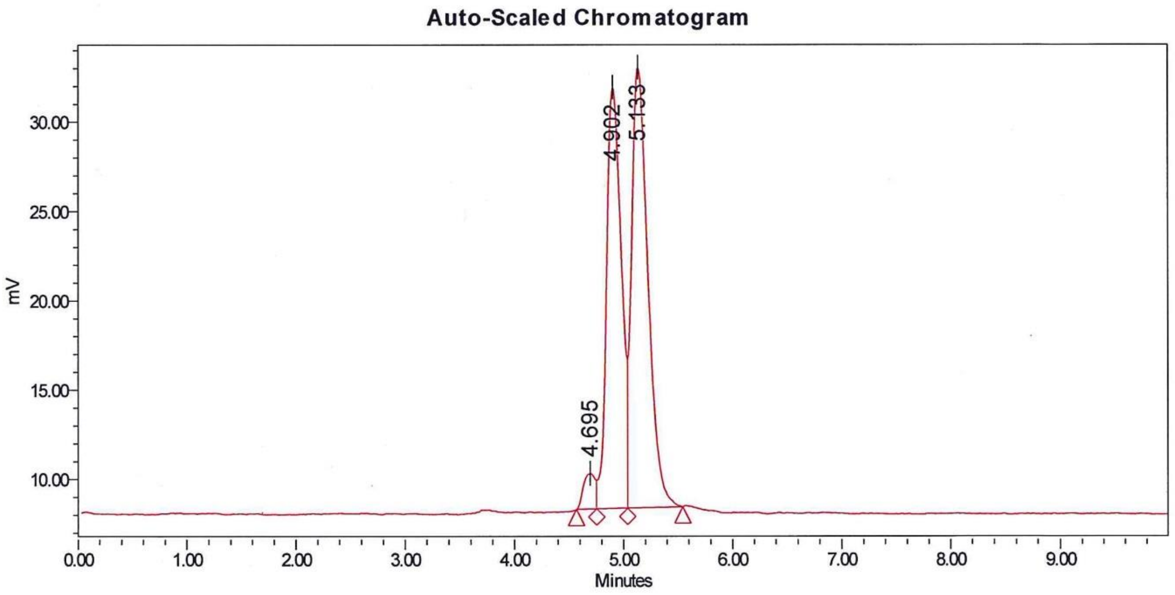

Supplementary Figure 9. Radio-HPLC chromatogram of zan-^{99m}Tc (after complexing with ^{99m}Tc). The radiochemical purity of ^{99m}Tc labeled zanamivir-chelate precursor (**compound 14**) was checked by radio-HPLC chromatography and found to be ~97% (as shown by the two major peaks in the chromatogram). No free ^{99m}Tc was detected.

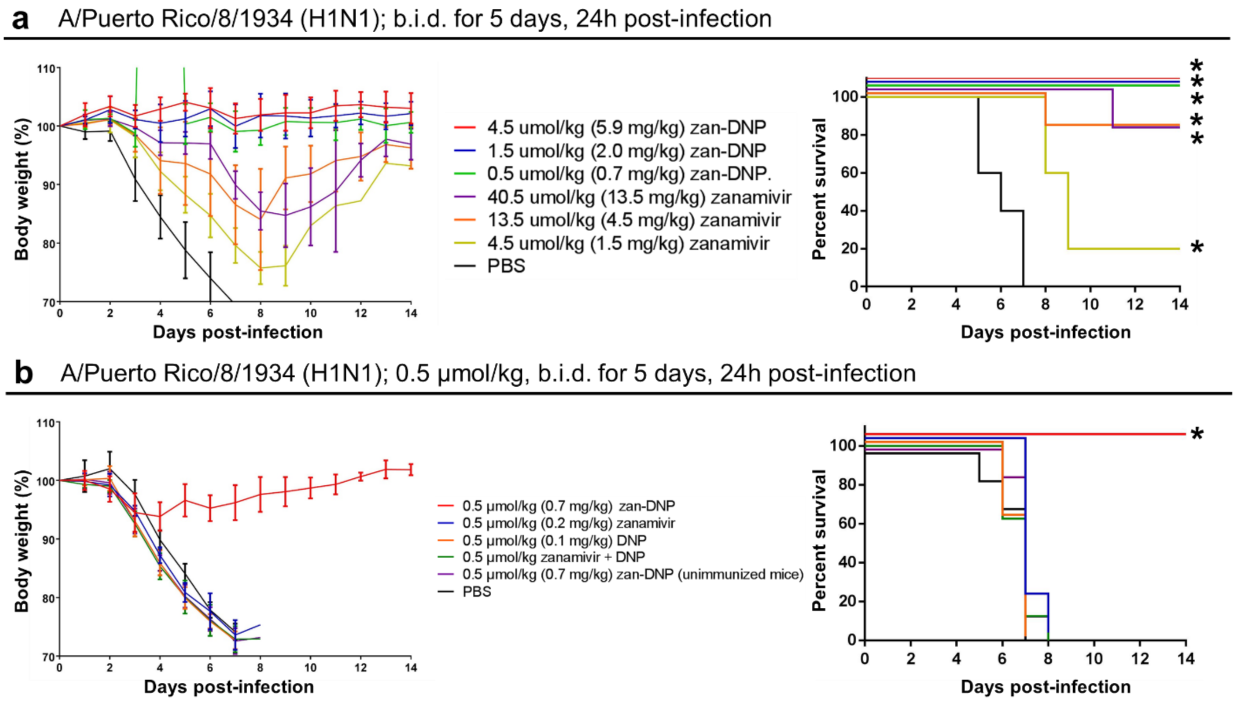

Supplementary Figure 10. In vivo therapeutic efficacy of zan-DNP following intraperitoneal administration. DNP-KLH immunized BALB/c mice were challenged with 100x MLD_{50} of influenza virus A/Puerto Rico/8/1934 (H1N1). Mice were treated with indicated test articles by intraperitoneal administration using the dosing regimens indicated on the figure. (**a**) Dose ranging study (5 mice/group). (**b**) Comparing the efficacy of zan-DNP with its component parts [5 mice/group for zan-DNP and zanamivir treatment groups, 8 mice/group for DNP and zanamivir + DNP treatment groups, 7 mice/group for zan-DNP (unimmunized mice) and PBS treatment groups]. Statistical differences between PBS and drug treatment groups were determined by two-sided log-rank test (*$P < 0.005$, see exact P values in Supplementary Table 1). Body weight change (%) are presented as mean values +/- SD.

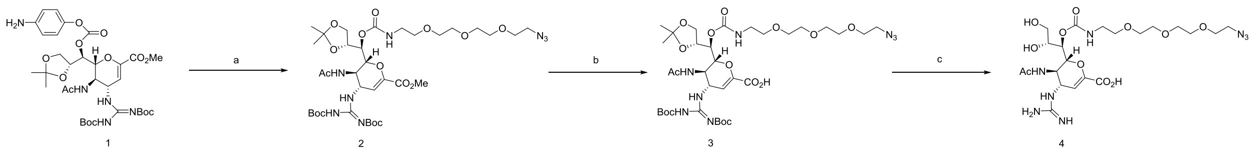

Supplementary Figure 11. Synthesis of zanamivir-related influenza neuraminidase targeting ligand. Reagents and conditions: (a) azido-dPEG®$_3$-amine, DMAP, pyridine; (b) 1M NaOH (aq), THF; (c) TFA.

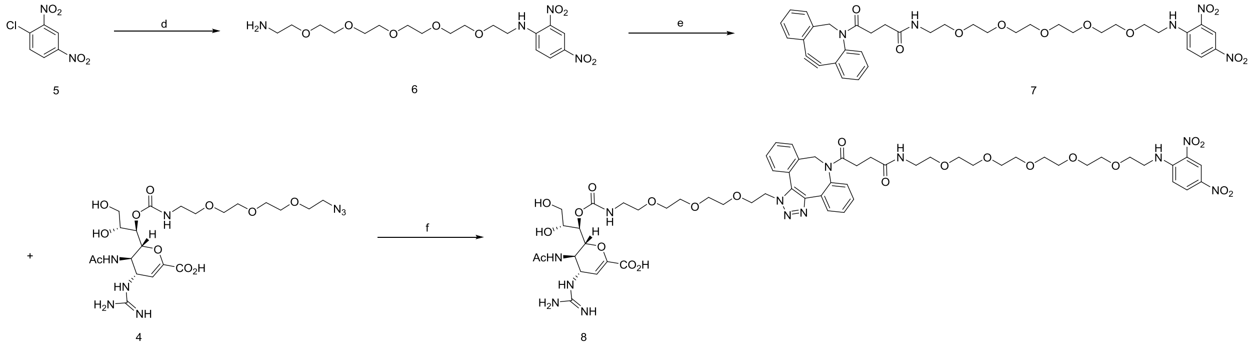

Supplementary Figure 12. Synthesis of zan-DNP. Reagents and conditions: (d) Amino-PEG$_5$-amine, TEA, EtOH; (e) DBCO-NHS, DIPEA, DMSO; (f) DMSO.

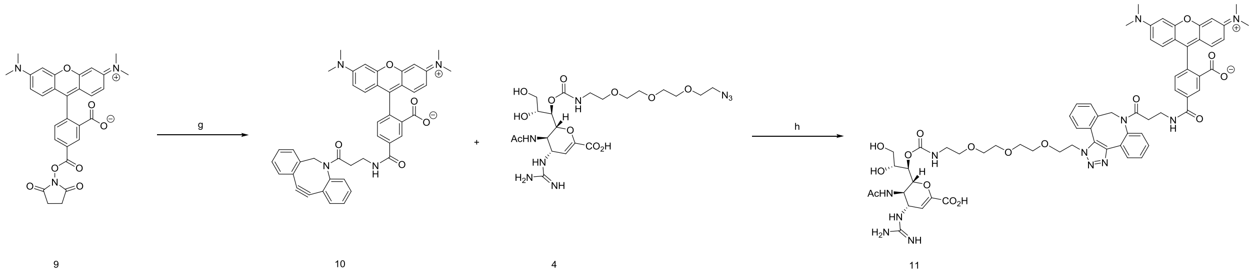

Supplementary Figure 13. Synthesis of zan-rhodamine. Reagents and conditions: (g) DBCO-amine, DIPEA, DMSO; (h) DMSO.

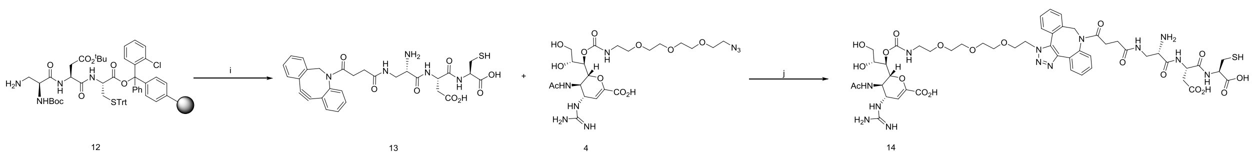

Supplementary Figure 14. Synthesis of zan-^{99m}Tc. Reagents and conditions: (i) 1. DBCO-acid, PyBop, DIPEA, DMF, 2. TFA/TIPS/EtSH/H_2O (92.5:2.5:2.5:2.5); (j) DMSO.

References

1 Chandler, M. *et al.* Synthesis of the potent influenza neuraminidase inhibitor 4-guanidino Neu5Ac2en. X-Ray molecular structure of 5-acetamido-4-amino-2, 6-anhydro-3, 4, 5-trideoxy-D-erythro-L-gluco-nononic acid. *Journal of the Chemical Society, Perkin Transactions 1*, 1173-1180 (1995).

2 Shidmoossavee, F. S., Watson, J. N. & Bennet, A. J. Chemical insight into the emergence of influenza virus strains that are resistant to Relenza. *Journal of the American Chemical Society* **135**, 13254-13257 (2013).

3 Ying, L. & Gervay ‐ Hague, J. One ‐ Bead ‐ One ‐ Inhibitor ‐ One ‐ Substrate Screening of Neuraminidase Activity. *ChemBioChem* **6**, 1857-1865 (2005).

4 Leamon, C. P. *et al.* Synthesis and biological evaluation of EC20: a new folate-derived, 99mTc-based radiopharmaceutical. *Bioconjugate chemistry* **13**, 1200-1210 (2002).

5 Lv, P.-C., Putt, K. S. & Low, P. S. Evaluation of nonpeptidic ligand conjugates for SPECT imaging of hypoxic and carbonic anhydrase IX-expressing cancers. *Bioconjugate chemistry* **27**, 1762-1769 (2016).
