# Supplementary material for: A universal dual mechanism immunotherapy for the treatment of influenza virus infections
Source: Nat Commun. 2020 Nov 5;11:5597. doi: 10.1038/s41467-020-19386-5 (PMC7645797; doi:10.1038/s41467-020-19386-5)
Supplement: Supplementary file 3 — Reporting Summary [file 41467_2020_19386_MOESM3_ESM.pdf]

nature research

Corresponding author(s): Philip S. Low

Last updated by author(s): Sep 10, 2020

Reporting Summary

Nature Research wishes to improve the reproducibility of the work that we publish. This form provides structure for consistency and transparency in reporting. For further information on Nature Research policies, see our Editorial Policies and the Editorial Policy Checklist.

Statistics

For all statistical analyses, confirm that the following items are present in the figure legend, table legend, main text, or Methods section.

n/a	Confirmed	
☐	☒	The exact sample size (*n*) for each experimental group/condition, given as a discrete number and unit of measurement
☐	☒	A statement on whether measurements were taken from distinct samples or whether the same sample was measured repeatedly
☐	☒	The statistical test(s) used AND whether they are one- or two-sided<br>*Only common tests should be described solely by name; describe more complex techniques in the Methods section.*
☒	☐	A description of all covariates tested
☒	☐	A description of any assumptions or corrections, such as tests of normality and adjustment for multiple comparisons
☐	☒	A full description of the statistical parameters including central tendency (e.g. means) or other basic estimates (e.g. regression coefficient) AND variation (e.g. standard deviation) or associated estimates of uncertainty (e.g. confidence intervals)
☐	☒	For null hypothesis testing, the test statistic (e.g. *F*, *t*, *r*) with confidence intervals, effect sizes, degrees of freedom and *P* value noted<br>*Give P values as exact values whenever suitable.*
☒	☐	For Bayesian analysis, information on the choice of priors and Markov chain Monte Carlo settings
☒	☐	For hierarchical and complex designs, identification of the appropriate level for tests and full reporting of outcomes
☒	☐	Estimates of effect sizes (e.g. Cohen's *d*, Pearson's *r*), indicating how they were calculated

Our web collection on statistics for biologists contains articles on many of the points above.

Software and code

Policy information about availability of computer code

Data collection	We used the instruments' built-in software for the data collection: Synergy Neo2 HTS MultiMode Microplate Reader (Biotek); FV 1000, Olympus confocal microscope; Attune™ NxT Flow Cytometer (Thermo Fisher Scientific); CFX Connect Real-Time PCR Detection System; γ-counter (Packard); micro-SPECT II/CT (MILabs). We did not design any new software for our studies.
Data analysis	GraphPad Prism 7 (GraphPad Software, CA); ImageJ; Microsoft Excel;

For manuscripts utilizing custom algorithms or software that are central to the research but not yet described in published literature, software must be made available to editors and reviewers. We strongly encourage code deposition in a community repository (e.g. GitHub). See the Nature Research guidelines for submitting code & software for further information.

Data

Policy information about availability of data

All manuscripts must include a data availability statement. This statement should provide the following information, where applicable:

- Accession codes, unique identifiers, or web links for publicly available datasets
- A list of figures that have associated raw data
- A description of any restrictions on data availability

All data are present in the main text and supplementary information or available from the authors on reasonable request. Source data are provided with this paper.

Field-specific reporting

Please select the one below that is the best fit for your research. If you are not sure, read the appropriate sections before making your selection.

☒ Life sciences ☐ Behavioural & social sciences ☐ Ecological, evolutionary & environmental sciences

For a reference copy of the document with all sections, see nature.com/documents/nr-reporting-summary-flat.pdf

Life sciences study design

All studies must disclose on these points even when the disclosure is negative.

Sample size	The sample size was chosen based on similar studies perfomed in studies published by other labs. We typically used 3 samples/group for the in-vitro assays and 5 mice/group for the in-vivo assays. The sample size for each experiment is indicated in the figure legend.
Data exclusions	No data were excluded.
Replication	All studies presented in the manuscript are reproducible. We tested our therapy using multiple different viral strains and different concentrations of drug. We included sufficient control groups in both in-vitro and in-vivo studies to avoid any potential false-positive or false-negative results.
Randomization	The animals were randomly allocated in each experimental groups according to their body weights before the start of experiments.
Blinding	Not performed.

Reporting for specific materials, systems and methods

We require information from authors about some types of materials, experimental systems and methods used in many studies. Here, indicate whether each material, system or method listed is relevant to your study. If you are not sure if a list item applies to your research, read the appropriate section before selecting a response.

Materials & experimental systems

n/a	Involved in the study	
☐	☒	Antibodies
☐	☒	Eukaryotic cell lines
☒	☐	Palaeontology and archaeology
☐	☒	Animals and other organisms
☒	☐	Human research participants
☒	☐	Clinical data
☒	☐	Dual use research of concern

Methods

n/a	Involved in the study	
☒	☐	ChIP-seq
☐	☒	Flow cytometry
☒	☐	MRI-based neuroimaging

Antibodies

Antibodies used

anti-DNP IgG-biotin conjugate (Invitrogen, cat. no. A-6435);
human anti-DNP IgG1 (ACROBiosystems, cat. no. DNP-M2);
goat anti-human F(ab')2-PE (Abcam, cat. no. ab98596);
rabbit antihemagglutinin antibody (Sino Biological Inc, cat. no. 86001-RM01);
goat anti-rabbit IgG-AF647 (Invitrogen, cat. no. A-21244);
rabbit anti-DNP IgG (Invitrogen, cat. no. A-6430);
goat anti-human IgG-HRP (Invitrogen, cat. no. 31410);
goat anti-human IgM-HRP (Invitrogen, cat. no. 31415);
goat anti-mouse IgG-HRP (Invitrogen, cat. no. 31430);
goat anti-mouse IgM-HRP (Invitrogen, cat. no. 31440);
rat anti-DNP antibodies (Invitrogen, cat. no. 04-8300)
goat anti-rat IgG-PE (Invitrogen, cat. no. A10545);

Validation	All antibodies are commercially available. The validation statements of the antibodies can be found from the manufacturers' websites:<br><br>anti-DNP IgG-biotin conjugate (Invitrogen, cat. no. A-6435):<br>https://www.thermofisher.com/antibody/product/Dinitrophenyl-KLH-Antibody-Polyclonal/A-6435;<br>human anti-DNP IgG1 (ACROBiosystems, cat. no. DNP-M2):<br>https://www.acrobiosystems.com/P1996-Monoclonal-Anti-DNP-%28Anti-Hapten%29-antibody-Human-IgG1.html;<br>goat anti-human F(ab')2-PE (Abcam, cat. no. ab98596):<br>https://www.abcam.com/goat-fab2-human-igg-fc-pe-pre-adsorbed-ab98596.html;<br>rabbit antihemagglutinin antibody (Sino Biological Inc, cat. no. 86001-RM01):<br>https://www.sinobiological.com/antibodies/hemagglutinin-ha-86001-rm01;<br>goat anti-rabbit IgG-AF647 (Invitrogen, cat. no. A-21244):<br>https://www.thermofisher.com/antibody/product/Goat-anti-Rabbit-IgG-H-L-Cross-Adsorbed-Secondary-Antibody-Polyclonal/A-21244;<br>rabbit anti-DNP IgG (Invitrogen, cat. no. A-6430):<br>https://www.thermofisher.com/antibody/product/Dinitrophenyl-KLH-Antibody-Polyclonal/A-6430;<br>goat anti-human IgG-HRP (Invitrogen, cat. no. 31410):<br>https://www.thermofisher.com/antibody/product/Goat-anti-Human-IgG-H-L-Secondary-Antibody-Polyclonal/31410;<br>goat anti-human IgM-HRP (Invitrogen, cat. no. 31415):<br>https://www.thermofisher.com/antibody/product/Goat-anti-Human-IgM-Secondary-Antibody-Polyclonal/31415;<br>goat anti-mouse IgG-HRP (Invitrogen, cat. no. 31430):<br>https://www.thermofisher.com/antibody/product/Goat-anti-Mouse-IgG-H-L-Secondary-Antibody-Polyclonal/31430;<br>goat anti-mouse IgM-HRP (Invitrogen, cat. no. 31440):<br>https://www.thermofisher.com/antibody/product/Goat-anti-Mouse-IgM-Secondary-Antibody-Polyclonal/31440;<br>rat anti-DNP antibodies (Invitrogen, cat. no. 04-8300):<br>https://www.thermofisher.com/antibody/product/DNP-Antibody-clone-LO-DNP-2-Monoclonal/04-8300;<br>goat anti-rat IgG-PE (Invitrogen, cat. no. A10545):<br>https://www.thermofisher.com/antibody/product/Goat-anti-Rat-IgG-H-L-Cross-Adsorbed-Secondary-Antibody-Polyclonal/A10545;

Eukaryotic cell lines

Policy information about cell lines

Cell line source(s)	Madin-Darby canine kidney (MDCK) cells and Human embryonic kidney 293 (HEK 293) cells were obtained from American Type Culture Collection (ATCC). Normal human bronchial epithelial (NHBE) cells were purchased from Lonza.
Authentication	All cell lines are commercially available and validation was performed by manufacturers.
Mycoplasma contamination	MDCK and HEK 293 cells were tested negative for mycoplasma contamination. NHBE cells were tested negative for mycoplasma contamination by the manufacturer. We used NHBE cells directly upon the arrival of the cells without performing the test.
Commonly misidentified lines<br>(See ICLAC register)	No commonly misidentified cell lines were used.

Animals and other organisms

Policy information about studies involving animals; ARRIVE guidelines recommended for reporting animal research

Laboratory animals	Female BALB/c mice (6 to 9 week old or 3 to 4 week old) were purchased from Envigo.
Wild animals	No wild animals were used in this study
Field-collected samples	No field-collected samples were used in this study
Ethics oversight	All animal procedures were approved by Purdue Animal Care and Use Committee (Protocol number: 1610001484).

Note that full information on the approval of the study protocol must also be provided in the manuscript.

Flow Cytometry

Plots

Confirm that:

- [x] The axis labels state the marker and fluorochrome used (e.g. CD4-FITC).
- [x] The axis scales are clearly visible. Include numbers along axes only for bottom left plot of group (a 'group' is an analysis of identical markers).
- [x] All plots are contour plots with outliers or pseudocolor plots.
- [x] A numerical value for number of cells or percentage (with statistics) is provided.

Methodology

Sample preparation	The lungs of virus-infected/uninfected mice were harvested immediately after euthanasia. The right lungs were digested using gentleMACS Octo Dissociator (Miltenyi Biotec) with mouse lung dissociation kit (Miltenyi Biotec, cat. no. 130-095-927). The cell suspensions were then filtered through 70 µm cell strainer (Miltenyi Biotec) and washed 2x with PBS. Erythrocytes were then removed using red cell lysis buffer (Biolegend) followed by PBS wash.
Instrument	Attune™ NxT Flow Cytometer (Thermo Fisher Scientific)
Software	We used this flow cytometer's build-in software (Attune™ NxT Software v3.1.2) to collect and analyze the data.
Cell population abundance	The cells were digested from the mouse lungs. No cell sorting was performed.
Gating strategy	Please see the detailed description in Figure 3E from the manuscript.

- [x] Tick this box to confirm that a figure exemplifying the gating strategy is provided in the Supplementary Information.
